# Prospective effects of mindfulness on anxiety and depressive symptoms may be spurious: Simulated reanalysis of a meta-analytic cross-lagged panel analysis

**DOI:** 10.1371/journal.pone.0302141

**Published:** 2024-05-13

**Authors:** Kimmo Sorjonen, Bo Melin

**Affiliations:** Department of Clinical Neuroscience, Karolinska Institutet, Stockholm, Sweden; St John’s University, UNITED STATES

## Abstract

A recent meta-analysis claimed decreasing prospective effects of acting with awareness and non-reacting, two facets of dispositional mindfulness, on subsequent anxiety and depressive symptoms. However, the meta-analytic cross-lagged effects were estimated while adjusting for a prior measurement of the outcome variable and it is known that such adjusted cross-lagged effects may be spurious due to correlations with residuals and regression to the mean. We fitted competing models on simulations of the same meta-analytic data and found that prospective effects of the mindfulness facets on anxiety and depressive symptoms probably were spurious. It is important for researchers to be aware of limitations of adjusted cross-lagged effects, meta-analytically estimated or not, in order not to overinterpret findings.

## Introduction

Mental ill-health, including depression, is a leading cause of disability worldwide [e.g. 1]. Consequently, it is of utmost importance to identify factors that may protect against mental ill-health. Using meta-analytic structural equation modeling, Prieto-Fidalgo et al. [2] found negative cross-lagged effects of initial acting with awareness and non-reacting, two facets of dispositional mindfulness, on subsequent anxiety and depressive symptoms when adjusting for initial anxiety and depressive symptoms, respectively. Prieto-Fidalgo et al. concluded that acting with awareness and non-reacting predicted subsequent decrease in symptoms.

However, it is known that cross-lagged effects while adjusting for a prior measurement of the outcome variable may be spurious due to correlations with residuals and regression to the mean [3–6]. As an example, let us assume that firemen tend to bake less than bakers. Consequently, if a fireman and a baker has baked equally much a particular week, we may suspect that the fireman has baked more than usually, i.e. experienced a positive residual, or that the baker has baked less than usually, i.e. experienced a negative residual. However, residuals tend to regress toward a mean value of zero between measurements. Consequently, we should expect a more negative change in amount of baking to the subsequent week for the fireman compared with the baker. With data from several individuals, we should expect a negative, but spurious, effect of a dichotomous “fireman vs. baker” variable on subsequent baking when adjusting for initial baking, even if no group-level change has taken place. Furthermore, as regression to the mean is independent of the direction of time, if the effect is spurious, we should expect a negative effect of “fireman vs. baker” on initial baking when adjusting for subsequent baking.

The objective of the present study was to reanalyze simulations of the meta-analytic data used by Prieto-Fidalgo et al. [2]. We did this in order to evaluate if the prospective adjusted effects of acting with awareness and non-reacting, two facets of dispositional mindfulness, on anxiety and depressive symptoms may have been spurious due to correlations with residuals and regression to the mean rather than, as suggested by Prieto-Fidalgo et al., truly decreasing. A wider objective was to show that adjusted cross-lagged effects, meta-analytically estimated or not, may often be spurious and should not be overinterpreted.

## Method

We refer to Prieto-Fidalgo et al. [2] for more comprehensive information on selection of studies, descriptive statistics, etc. In short, Prieto-Fidalgo et al. extracted sample sizes and zero-order correlations between initial dispositional mindfulness, initial anxiety or depressive symptoms, and subsequent anxiety or depressive symptoms from *k* = 34 studies. For the four combinations of facets of dispositional mindfulness and symptoms of mental ill-health among adults reanalyzed here, the number of included studies and sample sizes were: (1) acting with awareness and anxiety: *k* = 17, *N* = 1,175; (2) acting with awareness and depression: *k* = 24, *N* = 2,409; (3) non-reacting and anxiety: *k* = 13, *N* = 495; (4) non-reacting and depression: *k* = 16, *N* = 1,788.

We extracted 4 × 3 = 12 meta-analytic zero-order correlations between mindfulness and initial and subsequent symptoms, as well as the four total sample sizes, from Prieto-Fidalgo et al. Using the mvrnorm-function in the MASS package [7], we simulated four datasets with three variables each with these correlations and sample sizes. In each of the four simulated datasets we estimated: (1) The effect of initial mindfulness on subsequent symptoms while adjusting for initial symptoms. Both a hypothesis of a true decreasing effect and a hypothesis of a spurious effect due to correlations with residuals and regression to the mean predicted this effect to be negative; (2) The effect of initial mindfulness on initial symptoms while adjusting for subsequent symptoms. A hypothesis of a true decreasing effect predicted this effect to be positive, which would mean that among individuals with the same subsequent symptom score, those with a high initial mindfulness score had had a higher initial symptom score and, consequently, experienced a larger decrease in symptoms between measurements compared with those with the same subsequent symptom score but with a lower initial mindfulness score. Contrarily, as regression to the mean is independent of the direction of time, a hypothesis of spuriousness predicted this effect to be negative; (3) The effect of initial mindfulness on the subsequent symptoms–initial symptoms difference. A hypothesis of a true decreasing effect predicted this effect to be negative. Eq 1 gives the predicted effect of X on a Y2-Y1 difference [8]. Consequently, a hypothesis of spuriousness predicted this effect to be either close to zero (if the concurrent, *r*_*X*,*Y1*_, and cross-lagged, *r*_*X*,*Y2*_, correlations were approximately equally strong) or positive (if the concurrent correlation was stronger than the cross-lagged correlation).

$$E|\beta_{X1,Y2-Y1}|=\frac{r_{X1,Y2}-r_{X1,Y1}}{\sqrt{2(1-r_{Y1,Y2})}}$$ (1)

Simulations and analyses were conducted with R 4.1.3 statistical software [9] employing the MASS [7] and lavaan [10] packages. The analytic script, which also generates the simulated data, is available at the Open Science Framework at https://osf.io/bpc5z/.

## Results

**Fig 1:**
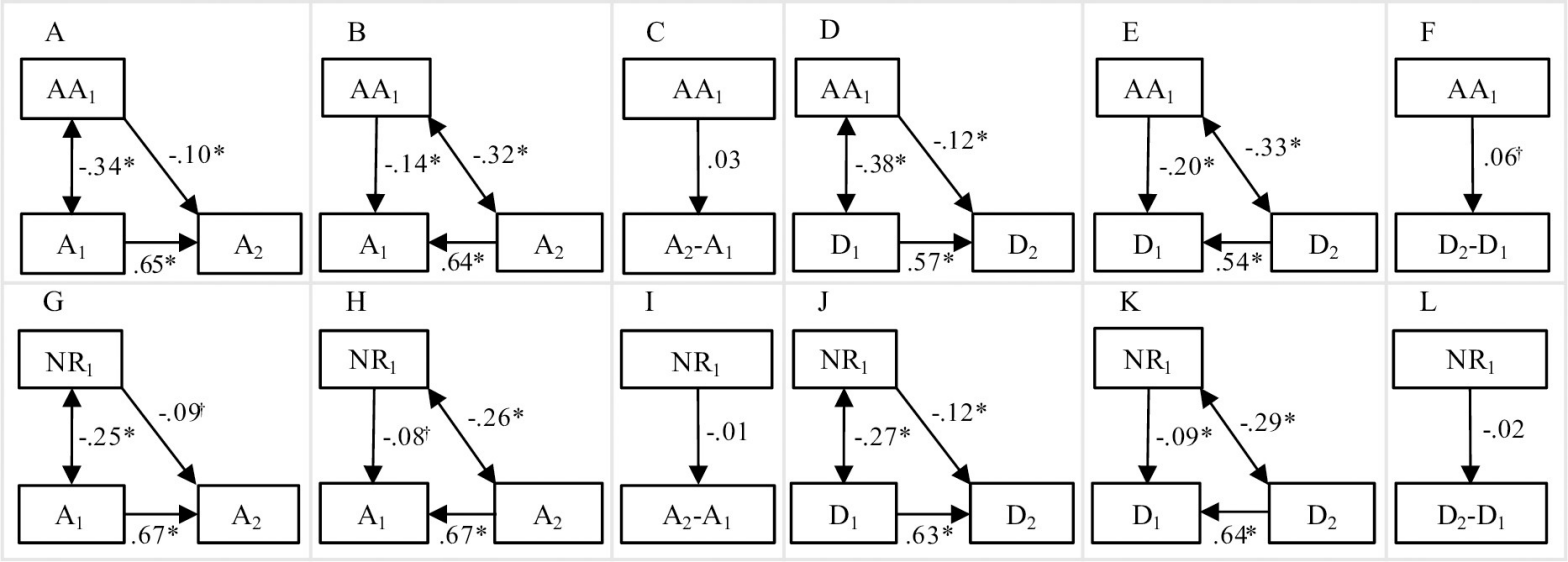
Regression effects and correlations in the models. Standardized regression effects and correlations in models with acting with awareness (AA, panels A-F) and non-reacting (NR, panels G-L) as predictors of anxiety (A, panels A-C and G-I) and depressive symptoms (D, panels D-F and J-L) measured at two occasions. Separately for models predicting subsequent anxiety/depressive symptoms while adjusting for initial anxiety/depressive symptoms (panels A, D, G, and J), models predicting initial anxiety/depressive symptoms while adjusting for subsequent anxiety/depressive symptoms (panels B, E, H, and K), and models predicting crude change (panels C, F, I, and L). * *p* < 0.001, ^†^ *p* < 0.05.

**Fig 2:**
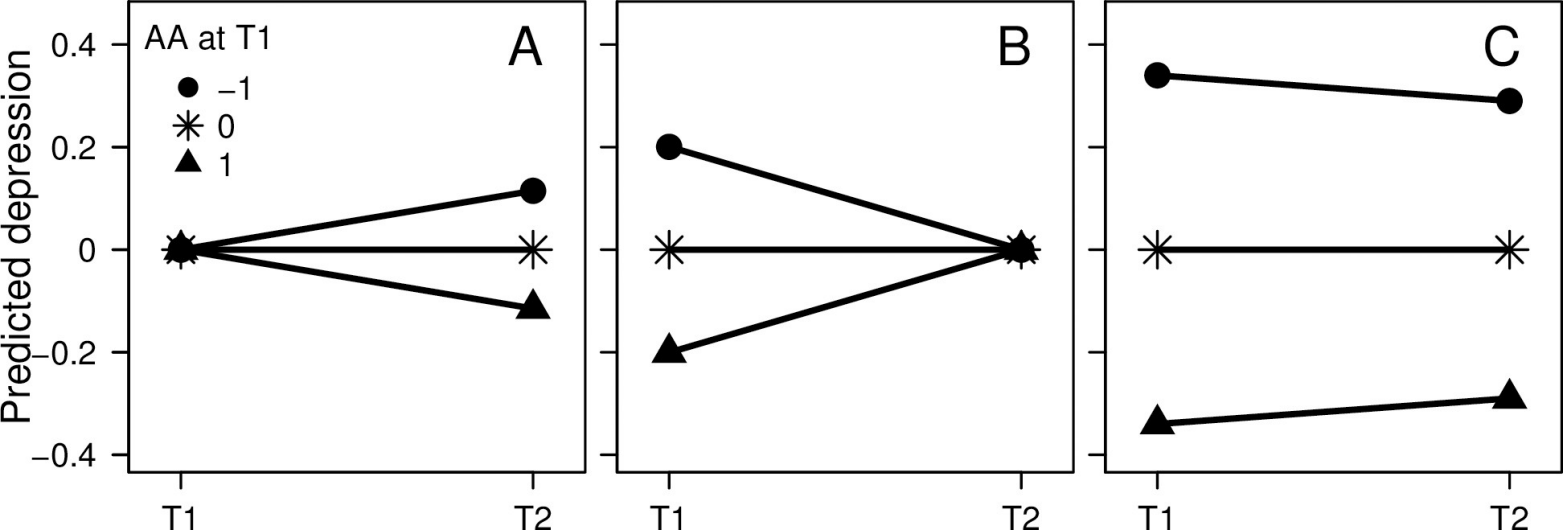
Predicted depressive symptoms. Predicted standardized initial and subsequent depressive symptoms for those with high (*Z* = 1), average, and low (*Z* = -1) initial acting with awareness (AA) when conditioning on average initial depressive symptoms (A), average subsequent depressive symptoms (B), and when not conditioning on depressive symptoms (C).

Effects in the three models for the four combinations of facet of mindfulness (acting with awareness and non-reacting) and outcome (anxiety and depressive symptoms) are presented in Fig 1. As already shown by Prieto-Fidalgo et al. [2], effects of initial mindfulness on subsequent symptoms while adjusting for initial symptoms were negative and statistically significant (Fig 1A, 1D, 1G and 1J). This suggests that a high initial score on mindfulness predicted a subsequent decrease in symptoms (illustrated with the effect of acting with awareness on depressive symptoms in Fig 2A). However, effects of mindfulness on initial symptoms while adjusting for subsequent symptoms were also negative and statistically significant (Fig 1B, 1E, 1H and 1K), indicating a larger subsequent increase in symptoms for those with a high initial mindfulness score compared with those with a lower initial mindfulness score (Fig 2B). Crude effects of initial mindfulness on the subsequent symptoms–initial symptoms differences were either non-significant (Fig 1C, 1I and 1L) or positive (Fig 1F), which would, again, mean a larger subsequent increase in symptoms for those with a high initial mindfulness score compared with those with a lower initial mindfulness score. The effects agreed better with a hypothesis of spurious prospective effects due to correlations with residuals and regression to the mean than with a hypothesis of true decreasing effects.

## Discussion

In the present reanalyses we found, as already shown by Prieto-Fidalgo et al. [2], negative meta-analytic effects of initial acting with awareness and non-reacting, two facets of dispositional mindfulness, on subsequent anxiety and depressive symptoms when adjusting for initial anxiety and depressive symptoms, respectively. This suggested decreasing effects of mindfulness on subsequent symptoms. However, we also found negative effects of initial mindfulness on initial symptoms when adjusting for subsequent symptoms, which indicated an increasing effect of mindfulness on subsequent symptoms, and either no or a positive crude effect of initial mindfulness on the subsequent symptoms–initial symptoms difference. These discrepant findings of decreasing, increasing, and no effects suggested that the prospective effects of mindfulness on anxiety and depressive symptoms may have been spurious due to correlations with residuals and regression to the mean rather than, as suggested by Prieto-Fidalgo et al. [2], truly decreasing.

As an example, picture two individuals, A and B, with the same initial depressive symptoms score but with A having a higher initial mindfulness score. Due to a negative association between mindfulness and depressive symptoms, and less than perfect reliability in the measurement of depressive symptoms, we may assume that A has received a higher depressive symptoms score compared with her true depressive symptoms score, i.e. a more positive residual, compared with B. However, as residuals tend to regress toward a mean value of zero between measurements, we should expect a more negative, but spurious, subsequent change in depressive symptoms for A compared with B.

It is important to note that the discrepancy between our conclusions and those by Prieto-Fidalgo et al. [2] is not due to our simulated data being very different from the data used by them. Contrarily, when fitting the same models, we received the same results as they did, i.e. negative cross-lagged effects of acting with awareness and non-reacting on subsequent anxiety and depressive symptoms (Fig 1, panels A, D, G, and J; the effects are within 0.01 from the corresponding effects presented by Prieto-Fidalgo et al.). This indicates a high degree of similarity between our simulated data and the data used by Prieto-Fidalgo et al. Instead, the discrepant conclusions are due to the fact that we, differently from Prieto-Fidalgo et al., fitted competing models to the same data and observed contradictory decreasing, increasing, and null effects of acting with awareness and non-reacting on subsequent anxiety and depressive symptoms. These contradictory findings lead us to conclude that effects probably were spurious. As an analogy, imagine that a researcher observes a decrease in degree of depression among patients wearing an amulet and concludes that wearing an amulet alleviates depression. A second researcher might observe exactly the same decrease in depression but still disagree with the conclusion, because he/she has observed a similar decrease in depression among patients not wearing an amulet.

The present study is part of a series where we have reanalyzed meta-analytic cross-lagged panel analyses [11–19]. In most cases we have found the adjusted prospective effects to be spurious rather than, as claimed in the original meta-analyses, truly increasing or decreasing. This points at the importance for researchers to be aware of limitations of cross-lagged effects, and of correlations in general, in order not to overinterpret findings, something that appears to have happened to Prieto-Fidalgo et al. [2]. The continued output of findings from uncritical use of cross-lagged panel analyses, meta-analytic or not, suggests that knowledge of limitations of the method, although far from new, is lacking in the research community. Hence, a continued reiteration of these limitations is warranted. As we did in the present study, researchers are recommended to fit alternative models, with predictions both forward and backward in time as well as crude effects on subsequent change, in order to discriminate between possibly true and spurious prospective effects.

### Limitations

The present reanalyses shared some of the limitations of the original meta-analysis by Prieto-Fidalgo et al. [2]. For example, the measurements of dispositional mindfulness and anxiety and depressive symptoms might not have been optimal in all of the included studies. Furthermore, we did not estimate possibly moderating effects of sex and age composition of the samples, time lag between measurements, etc. However, it is important to bear in mind that such characteristics were constant across the analyzed models and could, consequently, not explain the paradoxical findings of simultaneous decreasing, increasing, and null effects of initial mindfulness on subsequent change in symptoms.

## Conclusions

The present reanalyses found that meta-analytic prospective effects of acting with awareness and non-reacting, two facets of dispositional mindfulness, on anxiety and depressive symptoms probably were spurious, probably due to correlations with residuals and regression to the mean, rather than truly decreasing. It is important for researchers to be aware of limitations of cross-lagged effects, meta-analytically estimated of not, in order not to overinterpret findings.
